# Production of Poly(3-hydroxybutyrate-*co*-3-hydroxyhexanoate) from CO_2_ via pH-Stat Jar Cultivation of an Engineered Hydrogen-Oxidizing Bacterium *Cupriavidus necator*

**DOI:** 10.3390/bioengineering10111304

**Published:** 2023-11-10

**Authors:** Kenji Tanaka, Izumi Orita, Toshiaki Fukui

**Affiliations:** 1 Faculty of Humanity-Oriented Science and Engineering, Kindai University, 11-6 Kayanomori, Iizuka-shi 820-8555, Japan; 2 School of Life Science and Technology, Tokyo Institute of Technology, 4259 Nagatsuta, Midori-ku, Yokohama 226-8501, Japan; iorita@bio.titech.ac.jp (I.O.); tfukui@bio.titech.ac.jp (T.F.)

**Keywords:** polyhydroxyalkanoate, PHBHHx, CO_2_, Cupriavidus necator, hydrogen-oxidizing bacterium, high cell density cultivation, gas fermentation

## Abstract

The copolyester of 3-hydroxybutyrate (3HB) and 3-hydoxyhexanoate (3HHx), PHBHHx, is a biodegradable plastic characterized by high flexibility, softness, a wide process window, and marine biodegradability. PHBHHx is usually produced from structurally related carbon sources, such as vegetable oils or fatty acids, but not from inexpensive carbon sources such as sugars. In previous studies, we demonstrated that engineered strains of a hydrogen-oxidizing bacterium, *Cupriavidus necator*, synthesized PHBHHx with a high cellular content not only from sugars but also from CO_2_ as the sole carbon source in the flask culture. In this study, the highly efficient production of PHBHHx from CO_2_ was investigated via pH-stat jar cultivation of recombinant *C. necator* strains while feeding the substrate gas mixture (H_2_/O_2_/CO_2_ = 80:10:10 *v*/*v*%) to a complete mineral medium in a recycled-gas, closed-circuit culture system. As a result, the dry cell mass and PHBHHx concentration with the strain MF01/pBPP-ccr_Me_J_Ac_-emd reached up to 59.62 ± 3.18 g·L^−1^ and 49.31 ± 3.14 g·L^−1^, respectively, after 216 h of jar cultivation with limited addition of ammonia and phosphate solutions. The 3HHx composition was close to 10 mol%, which is suitable for practical applications. It is expected that the autotrophic cultivation of the recombinant *C. necator* can be feasible for the mass production of PHBHHx from CO_2_.

## 1. Introduction

**Figure 1:**
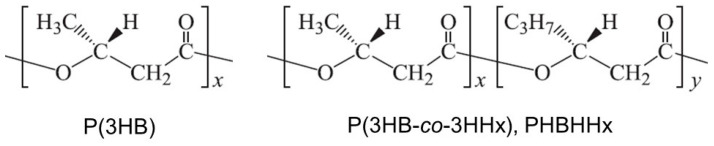
Chemical structures of P(3HB) and PHBHHx.

Bacterial polyesters, polyhydroxyalkanoates (PHAs), are environmentally friendly, biodegradable polymers, and are potential alternatives to petroleum-based polymeric materials. A homopolyester of (*R*)-3-hydroxybutyric acid, P(3HB), is well known to exhibit thermoplastic and biodegradable properties; therefore, P(3HB) was expected to be applicable in practical use. However, unfortunately, P(3HB) shows stiff and brittle properties [1] and has a very narrow “processing window” as the melting temperature (T_m_, 170–180 °C) is close to the thermal degradation temperature (180–190 °C) [2]. Copolyesters of 3HB and other hydroxyalkanoic acids (HAs) are candidates for more practical biodegradable polymer alternatives to P(3HB). In particular, a copolyester of 3HB and (*R*)-3-hydroxyhexanoic acid (3HHx), P(3HB-*co*-3HHx) (PHBHHx), is an attractive biodegradable plastic due to its superior thermal, mechanical, and physical properties [3]. The chemical structure of PHBHHx is shown in Figure 1.

In addition, bacterial PHAs are biodegradable not only in soil but also in marine environments [4] in contrast to many other biodegradable plastics, which show almost no biodegradability in such environments [5]. The increase in 3HHx units in PHBHHx decreases its crystallinity and melting temperature, leading to a wider processing window than those of P(3HB) and PHBV (the copolyester of 3HB and 3-hydroxyvaleric acid). This decrease in its melting point prevents the process temperature from reaching the thermal degradation temperature of the polymer. P(3HB-*co*-10 mol% 3HHx) shows some of the best properties for practical applications [6]. PHBHHx was originally found in *Aeromonas caviae* FA440 in 1993, although the cell growth and polymer accumulation of *A. caviae* were not very high [7]. Much research has been carried out to improve the biosynthesis of PHBHHx, for instance, by targeting its 3HHx content, molecular weight, and production efficiency, via several strategies and other bacteria, particularly *Cupriavidus necator* (the former names of this bacterium were *Alcaligenes eutrophus*, *Hydrogenomonas eutrophus*, *Ralstonia eutropha*, and *Wautersia eutropha*). The technology for PHBHHx production has progressed to a commercial scale. Kaneka Corporation (Tokyo, Japan) constructed a plant for PHBHHx production using recombinant *C. necator* from vegetable oil with a capacity of around 5000 tons/year in 2019 [8], and announced plans to expand the production up to 20,000 tons/year [9]. Most PHA copolymers, including PHBHHx, are produced from vegetable oils, fatty acids, or by adding precursor molecules that are structurally related to the second unit rather than 3HB during cultivation. These organic compounds are usually expensive in terms of raw materials in the fermentation industry; furthermore, such precursor compounds are often toxic to bacterial cells. The development of a method for efficient PHA production from an inexpensive carbon source is an urgent task for their integration into society [10]. Genetic engineering is one way to construct PHA copolymer biosynthesis pathways from structurally unrelated carbon sources. Many reports on the biosynthesis of 3HB-based copolymers from plant oils or fatty acids exist. In such cases, the (*R*)-3HA- coenzyme As (CoAs) were provided via β-oxidation of the acyl moieties [1]. Fukui and co-workers designed an artificial pathway for the biosynthesis of (*R*)-3HHx-CoA from sugar-derived acetyl-CoA molecules and synthesized it in *C. necator*. Their engineered strain, MF01*Δ*B1/pBPP-ccr_Me_J4a-emd, accumulated P(3HB-*co*-22 mol% 3HHx) within the cells at a high cellular content when it was cultured in a fructose medium [11]. They also created an engineered strain to accumulate P(3HB-*co*-11.7 mol% 3HHx) at a cellular content of 72 *w*/*w*% in a glucose medium [12].

*C. necator* is a hydrogen-oxidizing bacterium that can grow and accumulate P(3HB) under chemoautotrophic culture conditions provided with CO_2_ as the sole carbon source and H_2_ and O_2_ as the energy source and terminal electron acceptor, respectively. Among the known autotrophic organisms, *C. necator* shows high levels of specific growth rates, cell yield, and P(3HB) accumulation with CO_2_ [13]. Furthermore, *C. necator* has been the most well-studied bacterium for the biosynthesis of PHAs from organic compounds because it has a great ability to produce PHAs under heterotrophic conditions. In a previous study, we investigated the biosynthesis of PHBHHx using a flask culture of four engineered *C. necator* strains under autotrophic conditions provided with a substrate gas mixture of H_2_/O_2_/CO_2_ = 80:10:10 *v*/*v*% [14]. These recombinant strains accumulated PHBHHx with a high 3HHx composition and cellular content from CO_2_ as the sole carbon source. In particular, the strain MF01∆B1/pBBP-ccr_Me_J4a-emd synthesized the copolymer with a remarkably high 3HHx ratio of 47.7 ± 6.2 mol%. The strain MF01/pBPP-ccr_Me_J_Ac_-emd, harboring short-chain, length-specific (*R*)-enoyl-CoA hydratase (PhaJ), synthesized PHBHHx with a high cellular content, where the 3HHx composition was about 11 mol%, making it suitable for practical applications. The highest cell concentration (12.2 g·L^−1^) in the flask culture was obtained using MF01/pBPP-ccr_Me_J4a-emd. It was suspected that cell growth and PHBHHx accumulation in the flask culture ceased due to the decrease in pH.

In this study, we investigated the production of PHBHHx from CO_2_ in the pH-stat jar cultivation of the four recombinant strains of *C. necator* usialt medium (MS medium) was used for autotrophic cultng a jar fermenter and a recycled-gas, closed-circuit culture (RGCC) system for the chemoautotrophic cultivation. The cell growth, PHBHHx accumulation, and 3HHx levels in the resulting copolyesters were compared among these strains. The results showed that the concentration of cells and PHBHHx in the pH-stat jar cultivation increased up to about five times in comparison to the flask cultures. The production of PHBHHx from CO_2_ via an autotrophic culture of recombinant *C. necator* was comparable to heterotrophic production from an organic carbon source, such as plant oils, when the control of the gas composition in the RGCC system and the addition of inorganic nutrients during cultivation were further improved.

## 2. Materials and Methods

### 2.1. Bacterial Strains

The recombinant strains of *C. necator* used in this study were MF01/pBPP-ccr_Me_J4a-emd, MF01ΔB1/pBPP-ccr_Me_J4a-emd, MF01/pBPP-ccr_Me_J_Ac_-emd, and MF01ΔB1/pBPP-ccr_Me_J_Ac_-emd. The bacterial strains and plasmids used to create these recombinants are listed in Table 1. The construction of recombinant strains has been described in detail in previous reports [11,12].

### 2.2. Culture Medium

A mineral salt medium (MS medium) was used for autotrophic culture of the recombinant strains of *C. necator*. The composition of the MS medium used in the agar plate (subculture) and flask cultures (preculture) was 2.42 g NH_4_Cl, 4.0 g KH_2_PO_4_, 0.8 g NaHPO_4_, 1.0 g NaHCO_3_, and 0.2 g MgSO_4_·7H_2_O in 1 L of distilled water. These mineral salts except MgSO_4_·7H_2_O were dissolved in distilled water together while MgSO_4_·7H_2_O was independently dissolved in distilled water. The pH values of the mineral salt solution and the MgSO_4_·7H_2_O solution were separately adjusted to 7.0 then the two solutions were heated at 121 °C for 20 min. After cooling, they were aseptically mixed with the salt solution. A total of 0.1 mL of the trace element solution and 10 mL of a filter-sterilized kanamycin solution (20 mg/mL) were added to 1 L of the medium. The trace element solution was composed of 9.7 g FeCl_3_, 7.8 g CaCl_2_, 0.218 g CoCl_2_·6H_2_O, 0.118 g NiCl_3_·6H_2_O, 0.105 g CrCl_3_·6H_2_O, and 0.156 g CuSO_4_·5H_2_O per 1 L of 0.1 M HCl. 

### 2.3. Subculture and Preculture

All the strains were subcultured every 3 weeks on the MS plate medium containing 1.5 *w*/*w*% agar. The stock strain was inoculated onto the MS agar plates and placed in a desiccator equipped with a vacuum gauge. The air was evacuated using a vacuum pump and the inner space was filled with H_2_, O_2_, and CO_2_ in a ratio of 80:10:10 *v*/*v*%, respectively. The gas composition was adjusted by reading the vacuum gauge. The desiccator was placed at 30 °C for 72 h in an incubator for cell growth. These plate cultures were stored at 5 °C until the next subculture.

Preculture was autotrophically performed using 15 mL of the MS medium in a 300 mL Erlenmeyer flask for 72 h at a reciprocal shaking speed of 170 strokes/min to prepare the seed culture for jar cultivation. One loop of the cells was inoculated from the stock culture described above to the MS medium in the flask. Details of the flask culture under autotrophic conditions have been described previously [14,16].

### 2.4. Recycled-Gas, Closed-Circuit Culture System (RGCC Culture System)

**Figure 2:**
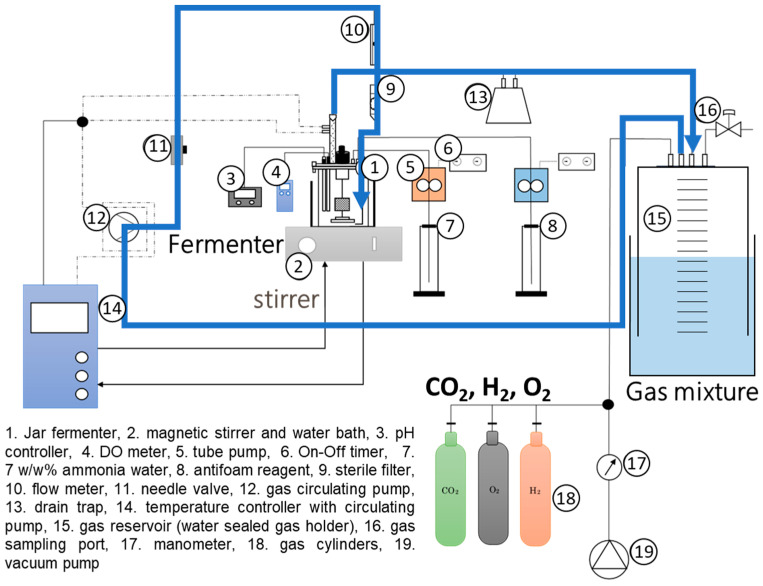
Schematic diagram of the RGCC culture system for the pH-stat jar cultivation of the hydrogen-oxidizing bacterium, *C. necator*, under chemoautotrophic conditions.

To investigate polymer production from CO_2_ employing wild-type and recombinant strains of *C. necator*, pH-stat jar cultivation was carried out using a recycled-gas, closed-circuit (RGCC) culture system. The RGCC system has been used in our previous studies [13,16,17,18,19,20,21,22] for the chemoautotrophic cultivation of hydrogen-oxidizing bacteria, as well as in a few other studies [23,24,25]. In the culture system, each substrate gas was introduced to a reservoir from its respective high-pressure gas cylinder. A schematic diagram of the culture system is shown in Figure 2. Detailed descriptions can be found in our previous reports [16,22]. 

A glass jar fermenter with a total volume of 1 L was used in this study in place of the 200 mL-scale micro jar fermenter used in our previous studies. The agitator unit was a two-blade-type magnetic stirrer bar with a rotating shaft. The volume coefficient of the mass transfer for oxygen (*K*_L_*a*) measured using the sulfite oxidation method was 250 h^−1^ at 900 rpm and 310 h^−1^ at 1300 rpm. A handmade water-sealed gas holder with a headspace capacity of 30 L was used as the new gas reservoir. The water-sealed gas holder was composed of two plastic buckets of different sizes. Four holes were provided at the bottom of the smaller bucket (total volume 45 L) and PTFE tubes were tightly inserted into all holes. The pipes were fixed with an adhesive material to prevent the gases from leaking out. They were connected to a vacuum pump, gas cylinders, gas sampling apparatus, and the culture system with silicone rubber tubes. This smaller bucket floated upside down in the larger bucket containing saturated salt water (total volume of 60 L). The air within the smaller bucket was evacuated with the vacuum pump before substrate gases were introduced from the cylinders and stored within the smaller bucket. The gas composition was adjusted to H_2_/O_2_/CO_2_ = 80:10:10 *v*/*v*% by supplying 32 L of H_2_, 4 L of O_2_, and 4 L of CO_2_ to the gas reservoir. The volume of the gases stored within the reservoir was measured by reading the depth to which the smaller bucket had sank into the saline reservoir via a ruler marked on the surface of the bucket. The gas mixture in the gas reservoir was fed to the MS medium in the fermenter using a diaphragm pump. The exhaust gas from the fermenter that was not absorbed in the medium was returned to the gas reservoir, and it was repeatedly fed to the fermenter to reduce the risk of detonation and the waste of substrate gases.

### 2.5. Conditions for the Main Culture

In the pH-stat jar cultivation, the MS medium with a low phosphate concentration (KH_2_PO_4_ 0.5 g·L^−1^ and NaHPO_4_ 0.1 g·L^−1^) was used. This was because a higher concentration of phosphate, added to prevent a decrease in pH, was found to reduce PHA accumulation in the flask culture. The working volume of the fermenter was 600 mL and the feed rate of the gas mixture to the fermenter was 600 mL·min^−1^ (equivalent to 1.0 vvm). The agitation speed was set to 900 rpm at the start of cultivation and was increased to 1300 rpm after 32 h. The pH was maintained at 7.0 by automatically adding a 7 *w*/*w*% ammonia solution with a pH controller (PHC-2201. Biott Co., Ltd., Tokyo, Japan). The gas composition within the reservoir was set to H_2_/O_2_/CO_2_ = 80:10:10 *v*/*v*% and it was reset to the initial ratio every 12 h by refilling the gases from the high-pressure cylinders. To prevent the culture broth from flowing out of the fermenter due to foaming, a diluted antifoaming reagent, A-nol (Biott), was added during cultivation.

### 2.6. Analyses

The cell concentration was monitored every 12 h by measuring the optical density at a wavelength of 600 nm (OD_600_) of the culture broth sampled from the fermenter. For determination of the dry cell mass (DCM) per 1 L, 10 mL of the culture broth was heated in a boiling water bath for 1 min, then centrifuged and washed with distilled water. The weight of the harvested cells was measured after drying at 105 °C for 24 h. The dissolved oxygen concentration (DO) was measured with a membrane-type DO sensor and DO meter (DJ-1033, Biott). The gas mixture was periodically extracted from the gas reservoir (the sample volume was 5 mL) and the composition was analyzed via gas chromatography (Shimadzu GC-8A) with a 4 mm × 6 m column, into which 5A molecular sieves and Porapack Q were packed. The content and monomer composition of PHBHHx accumulated within the cells were determined via gas chromatography. The cells were harvested via centrifugation from the heated culture broth and then lyophilized. The dried cells were subjected to direct methanolysis in methanol containing 15% sulfuric acid at 100 °C for 140 min. The resulting methyl esters of 3HB and 3HHx were quantified via gas chromatography, as described previously [26].

## 3. Results

### 3.1. pH-Stat Jar Cultivation of Recombinant Strains

**Figure 3:**
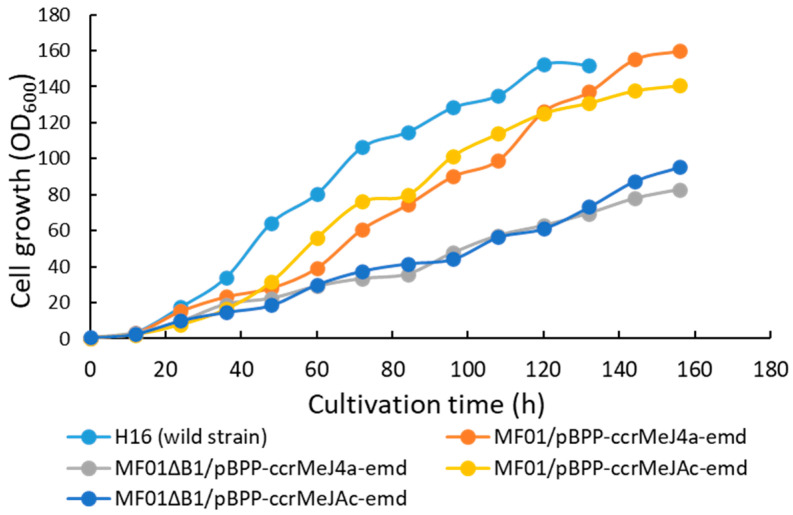
Time course for the increase in cell concentration (OD_600_) in the pH-stat jar cultivation of *C. necator* H16 and the recombinant strains under autotrophic conditions.

**Figure 4:**
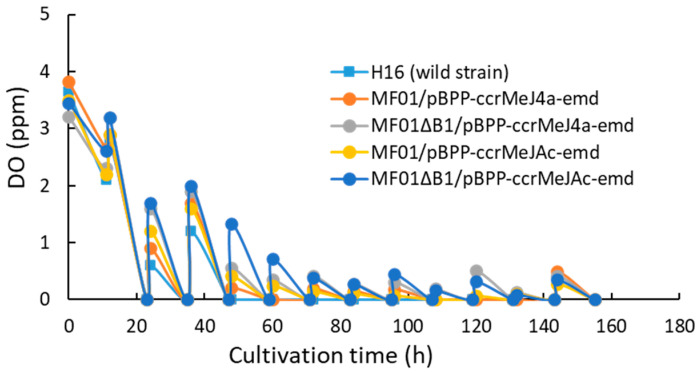
Time course for change in the dissolved oxygen concentration in the pH-stat jar cultivation of *C. necator* H16 and the recombinant strains under autotrophic conditions.

The *C. necator* wild-type strain H16 and four recombinant strains were cultivated in an MS medium containing 2.42 g·L^−1^ NH_4_Cl as a nitrogen source using an RGCC culture system by maintaining the pH at 7.0 with a 7 *w*/*w*% ammonia solution. The time courses of the cell concentration (OD_600_) and DO in the culture medium from the start until 156 h of cultivation are shown in Figure 3 and Figure 4, respectively. 

The growth of the recombinant strains was slower than that of H16. The specific growth rates of each strain from 12 h to 24 h were 0.159 h^−1^ for H16, 0.137 h^−1^ for MF01/pBPP-ccr_Me_J4a-emd, 0.094 h^−1^ for MF01ΔB1/pBPP-ccr_Me_J4a-emd, 0.107 h^−1^ for MF01/pBPP-ccr_Me_J_Ac_-emd, and 0.116 h^−1^ for MF01ΔB1/pBPP-ccr_Me_J_Ac_-emd. The exponential cell growth of strain H16 ceased at around 36 h. After that, the increase in cell concentration followed linear kinetics. Conversely, the exponential cell growth of the recombinant strains ceased between 36 h and 48 h, after which the increase in the cell concentration changed to linear.

The DO in the culture medium decreased to almost 0 ppm after 24 h, before cyclically increasing and decreasing again when the gas composition within the reservoir was reset to H_2_/O_2_/CO_2_ = 80:10:10 *v*/*v*%. The DO contents for all strains were always lower than 0.5 ppm after 72 h, which would promote polymer accumulation (Figure 4). 

**Figure 5:**
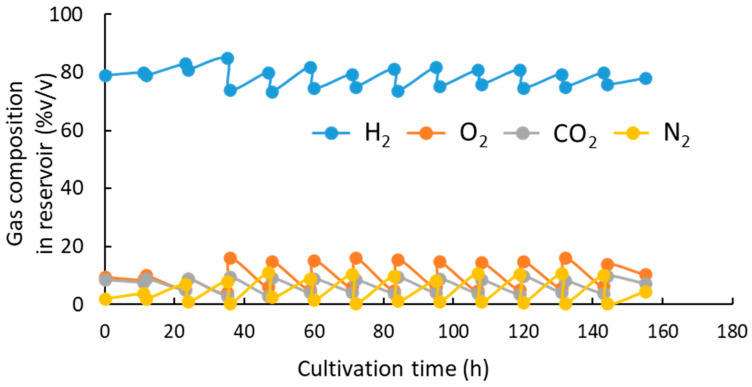
Change in gas composition within the gas reservoir in the pH-stat jar cultivation of *C. necator* MF01/pBPP-ccr_Me_J*_Ac_*-emd under autotrophic conditions.

The volume of the gas mixture within the reservoir (the water-sealed gas holder) decreased as the fermentation proceeded. The concentration of O_2_ and CO_2_ in the gas mixture within the reservoir drastically decreased while that of H_2_ increased a little (Figure 5). When the ratio of the substrate gases in the reservoir was well-balanced by that consumed by the cells, the volume of the gas mixture within the reservoir was expected to decrease without change in the gas composition. In the present pH-stat cultivation, the concentration of H_2_ within the reservoir increased, whereas those of O_2_ and CO_2_ decreased during the cultivation. This suggested a lower consumption of H_2_, as well as a higher demand for O_2_ and CO_2_, by the cells than that expected in the initial composition of the gases set at the start of the operation. The decrease in oxygen concentration in the gas mixture reflected the decrease in the DO concentration, which reduced the production rates of cells and polyester. An increase in the N_2_ concentration within the gas reservoir was also observed. This was probably due to the small volume of air remaining in the gas reservoir or mixing with the atmosphere during cultivation. Consequently, the concentration of N_2_ increased along with the consumption of the gas substrates within the reservoir. This led to the abovementioned resupplying of substrate gases to the reservoir every 12 h, whereby the gas composition was reset to the initial ratio. In the case of MF01/pBPP-ccr_Me_J*_Ac_*-emd, the volume of substrate gases consumed per 1 g of the produced cells was estimated to be about 9.1 L (H_2_), 2.4 L (O_2_), and 0.8 L (CO_2_) from the change in volume and composition of the gas mixture in the reservoir between 48 h and 72 h of cultivation. However, if gas leakage from the culture system occurred at a non-negligible level in this study, these estimations would not be reliable. The cellular consumption of the gas substrates will be precisely quantified in future studies.

Among the recombinant strains, the highest cell concentration at the end of cultivation was obtained for MF01/pBPP-ccr_Me_J4a-emd (OD_600_, 160.0), which was slightly higher than that of the wild-type strain H16. MF01/pBPP-ccr_Me_J_Ac_-emd also reached a high cell concentration. After cultivation, the cells were harvested from the culture broth and freeze-dried. The lyophilized cells were treated with methanolysis and subsequently analyzed via gas chromatography to determine their polymer content and monomer composition (Table 2). The highest content of PHBHHx in the cells (about 83.9 *w*/*w*%) was achieved by MF01/pBPP-ccr_Me_J_Ac_-emd. The highest 3HHx content (21.2 mol%) was obtained by MF01ΔB1/pBPP-ccr_Me_J4a-emd, while the 3HHx fraction in PHA synthesized by MF01/pBPP-ccr_Me_J_Ac_-emd was 10.9 mol%, which is very close to the favorable value for the practical use of PHBHHx. The monomer compositions of PHBHHx obtained in the pH-stat jar cultivation of the recombinant strains were comparable to those obtained by the corresponding flask culture with 1 g·L^−1^ of (NH_4_)_2_SO_4_, except in the case of MF01ΔB1/pBPP-ccr_Me_J4a-emd, which exhibited 47.7 mol% 3HHx [14]. In this study, pH-stat jar cultivation with the feeding of a 7 *w*/*w*% ammonia solution as the nitrogen source was performed once for each recombinant strain; thus, the culture test should be repeated to confirm these trends. We also observed that the 3HHx content in the pH-stat jar cultivation of MF01ΔB1/pBPP-ccr_Me_J4a-emd with the feeding of 3 M KOH instead of an ammonia solution was also lower than that seen in the flask culture. 

Table 3 shows the PHBHHx concentration and process productivity at the end of the pH-stat jar cultivation. The PHBHHx productivity in the pH-stat jar cultivation increased by up to about 3–4 times in comparison to the flask culture [14]. The highest productivity was obtained by MF01/pBPP-ccr_Me_J_Ac_-emd, although it was lower than that for the P(3HB) homopolyester obtained by the H16 strain.

### 3.2. pH-Stat Jar Cultivation of MF01/pBPP-ccr_Me_J_Ac_-emd with Supplemented Phosphate

**Figure 6:**
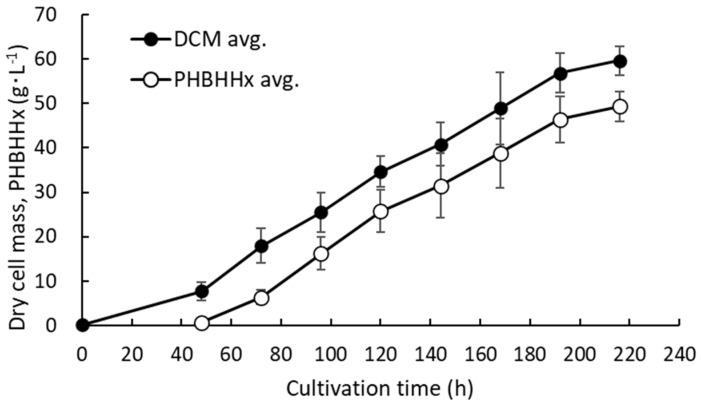
Time courses for the increase in DCM and PHBHHx in the pH-stat jar cultivation of MF01/pBPP-ccr_Me_J_Ac_-emd under autotrophic conditions with the addition of 0.2 g·L^−1^ of KH_2_PO_4_ to the culture medium at 96 h and 168 h. (*n* = 3).

MF01/pBPP-ccr_Me_J_Ac_-emd showed good results regarding the cell concentration, PHBH accumulation, and 3HHx content. Hence, to further increase the production of the cells and PHBHHx, the pH-stat jar cultivation was carried out with the addition of 0.2 g·L^−1^ of KH_2_PO_4_ in the culture medium at 96 h and 168 h. The culture experiment was carried out three times under the same conditions. Figure 6 shows the increase in dry cell mass (DCM) and PHBHHx concentration every 24 h during the cultivation. 

After 216 h, the average concentrations of DCM and PHBHHx increased to 59.62 ± 3.18 g·L^−1^ and 49.31 ± 3.14 g·L^−1^, respectively, while the final cellular PHBHHx content and the 3HHx content were 82.7 ± 1.37 *w*/*w*% and 9.3 ± 3.7 mol%, respectively. The NH_4_-N concentration in the supernatant of the culture medium was determined using the indophenol blue colorimetric method, and the PO_4_^3−^ concentration was also determined using the molybdenum blue colorimetric method. The NH_4_-N concentration increased to 1054 ± 72.5 mg·L^−1^ as soon as the ammonia solution was fed to the solution to prevent the decrease in pH caused by the supply of the gas mixture. After that, the NH_4_-N concentration decreased to about 119.1 ± 52.7 mg·L^−1^ at the end of the cultivation. The PO_4_^3−^ concentration decreased from 410 ± 18 mg·L^−1^ to an undetectable level at around 40 h. When KH_2_PO_4_ was supplemented, the PO_4_^3−^ concentration increased at once, but it was mostly consumed at the end of the cultivation.

The PHBHHx productivity was 0.228 ± 0.016 g·L^−1^·h^−1^, almost equal to that in the cultivation of the strain without phosphate supplementation. 

## 4. Discussion

There has been growing interest in PHA production from CO_2_ by autotrophic microorganisms, and the number of research reports relating to hydrogen-oxidizing bacteria is also increasing. For instance, there has been research focusing on fermentation processes to maintain the O_2_ concentration in the substrate gas below the lower limit for detonation [27,28,29,30], precise measurements of gas consumption and stoichiometry for PHA production [27,29,31,32], improvements in productivity [33], and the production of PHA copolymers. However, most studies on the production of PHA copolymers from CO_2_ examined a mixotrophic culture, where organic compounds structurally related to the secondary hydroxyacyl monomer units were added to the culture medium during the PHA accumulation period [25,34,35]. Although some researchers reported the biosynthesis of PHA copolymer from CO_2_ as a sole carbon source [14,28,36], the concentration of the resulting copolyesters was still very low. The PHBHHx concentration and process productivity that we obtained through a completely autotrophic culture of MF01/pBPP-ccr_Me_J_Ac_-emd in this study were much higher than those observed in previous related reports but were not always higher than the results obtained in mixotrophic cultivations.

The pathway for the biosynthesis of PHBHHx from CO_2_, containing the artificial pathway for the formation of 3HHx-CoA from acetyl-CoA, has been described in previous reports [11,14]. The monomer compositions of PHBHHx obtained in the pH-stat jar cultivation of the recombinant strains tended to be comparable to those obtained in the flask culture, suggesting that the PHBHHx biosynthesis pathway was not significantly affected by the culture conditions. One exception was the strain MF01ΔB1/pBPP-ccr_Me_J4a-emd, for which the 3HHx fraction in the autotrophically synthesized polymer was markedly decreased to 21.2 mol% when compared with the 47.4 mol% observed in the flask culture. In the case of the flask culture without any control, it was observed that the pH of the medium decreased during cultivation, and/or the dissolved concentrations of the gaseous substrates were different from those in the jar. As such, these altered statuses may affect the incorporation of the 3HHx unit into the polyester chains, although the details are yet to be elucidated. 

The inorganic nutrients contained in the MS medium are utilized for the biosynthesis of cell components such as proteins and nucleic acids and the activation of enzymes and other physiological functions in cells. While it is known that PHA accumulation is promoted by the limitation of inorganic nutrients (especially nitrogen (N)- and phosphorus (P)-containing compounds), this will lead to an imbalanced composition in relation to the carbon source in the culture medium. PHA accumulation in the autotrophic cultivation of hydrogen-oxidizing bacteria was also promoted by limiting N and/or P sources, while they were essential to achieve a high cell density leading to a high PHA concentration. Therefore, N and P sources and other mineral salts are required to be supplemented into the medium along with the carbon source during the cultivation to an extent that does not inhibit PHA accumulation. In the heterotrophic production of PHAs with the feeding of organic carbon sources, very high PHA concentrations are often obtained by limiting the feed amount of nitrogen [37] or phosphorus [38] compared to that of the carbon source during the PHA accumulation period. However, in the case of autotrophic cultivation, it is difficult to feed gaseous CO_2_ and other nutrients with a constant C/N or C/P ratio. In this study, an ammonia solution (N source) was gradually added by using a pH controller to keep the NH_4_ concentration at low levels, as well as to maintain the pH of the culture medium, because the decrease in pH during the cultivation was mainly due to the consumption of NH_4_^+^ and dissolution of CO_2_. A small amount of KH_2_PO_4_ solution (P source) was added twice during the PHA accumulation stage. This N- and P-feeding strategy resulted in the successful enhancement of PHBHHx production to a certain extent. However, the concentrations of NH_4_-N and PO_4_^3−^ still changed during the cultivation and they often significantly increased when the gas reservoir was refilled with new gases every 12 h. The concentrations of O_2_ and CO_2_ in the gas reservoir quickly increased to the initial levels after refilling the reservoir, as shown in Figure 5. The increase in CO_2_ concentration accelerated the addition of ammonia solution to maintain the pH of the culture medium. It may be possible to increase the cell biomass and PHBHHx beyond the maximum produced in this study by improving the feeding strategy of ammonia and K_2_HPO_4_ solutions to maintain constantly low concentrations of NH_4_ and PO_4_^3−^. It is also known that dissolved oxygen (DO) promotes PHA accumulation. However, the DO-limited PHBHHx synthesis in the autotrophic cultivation of *C. necator* was gradually suppressed when NH_4_-N and PO_4_^3−^ remained at a high concentration in the culture medium. Hence, our pH-stat jar cultivation was initiated in a culture medium containing a low phosphate concentration; then, an ammonia solution and KH_2_PO_4_ were gradually supplemented during the cultivation to support cell growth and PHBHHx accumulation. We investigated the influence of NH_4_-N and PO_4_^3−^ on PHBHHx biosynthesis by *C. necator* under the DO-limited conditions in detail.

The production of cells and PHA in the autotrophic culture depended on the mass transfer of the substrate gases in the culture medium. The oxygen transfer rate was particularly important because the DO limitation was inevitable at a high cell concentration. The O_2_ concentration in the gas phase for the cultivation of hydrogen-oxidizing bacteria was usually lower than that in air because most of these bacteria exhibit sensitivity to oxygen and their optimal O_2_ concentration is much lower than that that in the atmosphere. This is one of the reasons why we used the gas composition of H_2_/O_2_/CO_2_ = 80:10:10 *v*/*v*%. Furthermore, it was effective to keep the O_2_ concentration below the lower limit for explosion (around 4.0 vol%) to prevent detonation [19]. The oxygen transfer rate in the culture medium decreased according to the decline in oxygen concentration in the gas mixture, which diminished the production of cells and PHA [19]. Therefore, it is expected that the production of PHBHHx in the autotrophic culture of the recombinant strains of *C. necator* could be increased by improving the supply of the substrate gases into the reservoir to maintain the gas composition at an adequate constant ratio and by enhancing the mixing effect of the gases in the culture medium. We have already succeeded in increasing the cell growth and homopolyester P(3HB) production to 91.3 g·L^−1^ and 62 g·L^−1^, respectively, after 40 h of autotrophic cultivation of the wild strain H16 using a basket-shaped agitation unit [19].

As mentioned above, several points in the fermentation process are yet to be optimized to achieve more efficient production of PHBHHx from CO_2_. We are continuing this study to optimize ammonia solution and inorganic salt feeding, the stabilization of the gas composition, and the gas mass transfer by using a high-performance agitator in the fermenter to elevate the concentration and production of PHBHHx, which will be reported in the near future. 

## Figures and Tables

**Table 1:** Bacterial strains and plasmids used in this study.

Strain	Relevant Marker	References or Resources
*C. necator* H16	Wild type	DSM 428
*C. necator* MF01	H16 derivative; Δ*pha*C::*phaC_NSDG_*, Δ*phaA*::*bktB*	[15]
*C. necator* MF01ΔB1	MF01 derivative; Δ*phaB1*	[11]
Plasmid		
pBPP	pBBR1-MCS2 derivative; *P_phaP1_*, *T_rrnB_*	[15]
pBPP-ccr_Me_J4a-emd	pBPP derivative; *ccr_Me_*, *phaJ4a*, *emd_Mm_*	[11]
pBPP-ccr_Me_J_Ac_-emd	pBPP derivative; *ccr_Me_*, *phaJ_Ac_*, *emd_Mm_*	[12]

*phaA*, β-ketothiolase gene; *bktB*, broad-substrate-range β-ketothiolase gene; *phaB1*, NADPH-dependent acetoacetyl-CoA reductase gene; *phaC*, PHA synthase gene in the *pha* operon on chromosome 1; *phaC_NSDG_*, a gene of the N149S/D171G double mutant of broad-substrate-range PHA synthase from *Aeromonas cavaie*; *P_phaP1_*, promoter region of *phaP1*; *T_rrnB_*, transcription terminator region from *E. coli*; *ccr_Me_*, crotonyl-CoA carboxylase/reductase gene from *Methylorubrum extorquens*; *emd_Mm_*, a codon-optimized gene-encoding ethylmalonyl-CoA decarboxylase from *Mus musculus*; *phaJ4a,* medium-chain-specific (*R*)-2-enoyl-CoA hydratase gene; *phaJ_Ac_*, short-chain-specific (*R*)-2-enoyl-CoA hydratase gene from *A. caviae.*

**Table 2:** Cell growth and PHA accumulation in the autotrophic pH-stat jar cultivation of *C. necator* H16 and the recombinant strains.

Strain/Plasmid	Dry Cell Mass (g·L^−1^)	PHA Content in Cells (*w*/*w*%)	Monomer Composition (mol%)	
			3HB	3HHx
H16 (wild strain)	44.05	78.5	100.0	0
MF01/pBPP-ccr_Me_J4a-emd	45.42	57.7	92.9	7.1
MF01ΔB1/pBPP-ccr_Me_J4a-emd	23.06	66.8	78.8	21.2
MF01/pBPP-ccr_Me_J_Ac_-emd	40.12	83.9	89.1	10.9
MF01ΔB1/pBPP-ccr_Me_J_Ac-_emd	26.70	75.5	90.9	9.10

**Table 3:** PHA concentration and productivity of the autotrophic pH-stat jar cultivation of *C. necator* H16 and the recombinant strains.

Strain/Plasmid	Cultivation Time (h)	PHBHHx Concentration (g·L^−1^)	Productivity (g·L^−1^·h^−1^)
H16 (wild strain)	120	P(3HB) 34.58	P(3HB) 0.288
MF01/pBPP-ccr_Me_J4a-emd	156	26.19	0.168
MF01ΔB1/pBPP-ccr_Me_J4a-emd	156	15.40	0.099
MF01/pBPP-ccr_Me_J_Ac_-emd	156	33.66	0.216
MF01ΔB1/pBPP-ccr_Me_J_Ac-_emd	156	20.16	0.129

## Data Availability

Not applicable.
